# Impaction bone grafting for segmental acetabular defects: a biomechanical study

**DOI:** 10.1007/s00402-021-04296-y

**Published:** 2021-12-14

**Authors:** Wagener Nele, Fritsch Martina, Reinicke Stefan, Layher Frank, Matziolis Georg

**Affiliations:** 1 Orthopaedic Department of the Waldkliniken Eisenberg, Orthopaedic Professorship of the University Hospital Jena, 07607 Eisenberg, Germany; 2 Department of Trauma Surgery, Orthopaedics and Plastic Surgery, University Medical Center Göttingen, Georg-August-University, Robert Koch Straße 40, 37075 Göttingen, Germany

**Keywords:** Revision total hip arthroplasty, Bone defect, Impaction bone grafting, Biomechanical study, Migration, Torsional stiffness

## Abstract

**Introduction:**

Implant loosening is the most common indication for revision after total hip arthroplasty and is associated with progressive bone destruction. Contained defects can be treated with impaction bone grafting (IBG). Segmental defects are successfully restored with metal augmentation. Considering the increasing number of hip arthroplasty cases in young patients, it would appear sensible to reconstruct the bone stock for future revisions by biological bone defect reduction. The data on the treatment of segmental defects with IBG without additional stabilization are lacking.

**Materials and methods:**

Paprosky type IIB defects were milled into 15 porcine hemipelves with segmental defect angles of 40°, 80° and 120°. Contained defects without segmental defects (Paprosky type I) and acetabula without defects served as controls. After IBG, a cemented polyethylene cup (PE) was implanted in each case. Cup migration, rotational stiffness and maximum rupture torque were determined under physiological loading conditions after 2500 cycles.

**Results:**

Compared with the control without defects, IBG cups showed an asymptotic migration of 0.26 mm ± 0.11 mm on average. This seating was not dependent on the size of the defect. The maximum rupture moment was also not dependent on the defect size for cups after IBG. In contrast, the torsional stiffness of cups with an 120° segmental defect angle was significantly lower than in the control group without defects. All other defects did not differ in torsional stiffness from the control without defects.

**Conclusions:**

IBG did not show inferior biomechanical properties in segmental type IIB defect angles up to 80°, compared to cups without defects.

## Introduction

The greatest challenge in acetabular revision surgery is to achieve sufficient primary stability despite the regular presence of bone defects [1, 2]. Owing to the increasing number of young patients undergoing arthroplasty, an increase in revisions as well as a re-revision burden are to be expected [3]. Without allogeneic reconstructions, bone defects increase with each revision and complicate subsequent re-revision [4–6].

Craniolateral defects of the Paprosky IIB type, which are subject to the highest mechanical load, are regularly treated using metallic augments with or without support shells. In contrast, cavitary defects can be easily and successfully addressed with IBG. The main advantage of such a biological solution compared to metallic defect fillings is the build-up of a bone stock for future revisions, as IBG allows "down-grading" of bone defects. IBG is also described for segmental defects, but is performed in combination with support shells, meshes or structured bone grafts.

At present, no data are available on IBG for sole use on uncontained defects of the common type IIB, without the use of additional support shells, meshes, or structured bone grafts. The aim of the present biomechanical cadaver study was to investigate whether and up to which size segmental type IIB defects can be solely treated with IBG in order to achieve sufficient primary stability.

## Materials and methods

### Bone preparation

Fifteen porcine pelvic bones served as hip joint models. The cadaveric porcine pelvic bones were obtained from a local licensed abattoir post mortem. They were stored at − 20 °C. Thawing to room temperature took place 24 h before preparation of the hip joints. The pigs´ pelvises were fixed stably in a metal sleeve with a diameter of 90 mm, using pebbles and a resin mixture (Orthoacrylic sealing resin from Otto Bock). Rotational stability was achieved by additional transverse screwing. Cancellous bone chips were collected from femoral heads and condyles of pigs. Bone chips of 5 mm size were prepared using a bone mill. The bone chips were then washed three times to remove all bone marrow and fat. The bone chips were dried by centrifugation three times. This was followed by 2 hours of air drying. The bone chips were stored at − 20 °C until use [7, 8].

### Cup preparation

Segmental defect milling was performed on three porcine hemipelves, each with 40°, 80° and 120° segmental defect angles of Paprosky type IIB. In order to cover the entire craniolateral acetabular cavity area, we selected the segmental defect angles of 40°, 80° and 120° based on the Paprosky classification type IIB.

**Fig. 1:**
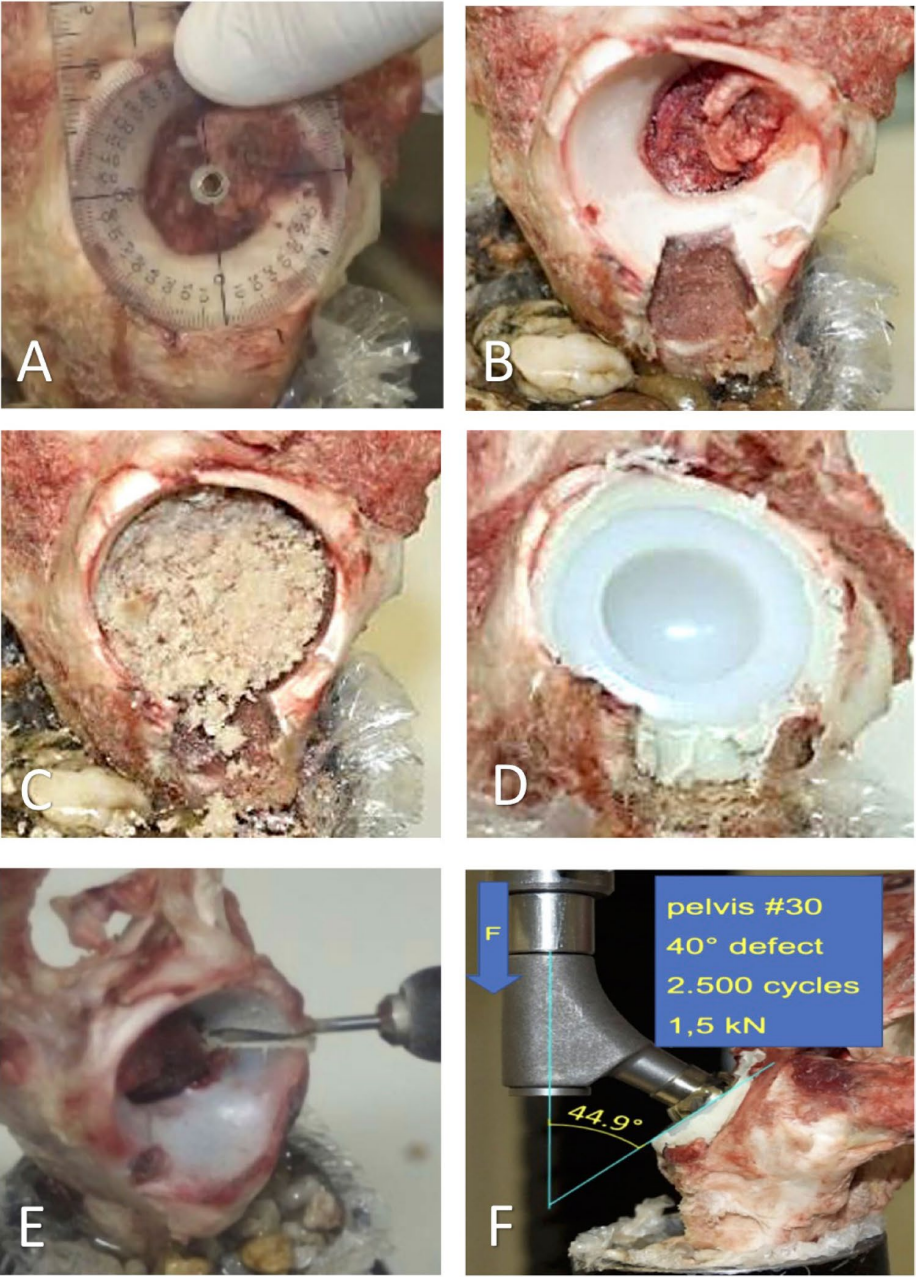
Shows the marking of the defect areas according to the corresponding segmental defect angles (**A**), fixation of a hemipelvis with a 40° segmental defect angle in the metal cylinder (**B**), followed by allogenic cancellous bone graft in a Paprosky type II segmental defect angle of 40° (**C**), impaction and cemented cup implantation (**D**) and the milling of a 120° segmental defect angle (**E**). Clamping a specimen with a segmental defect angle of 40° into the biotesting machine. The arrow F indicates the direction in which the force acts (**F**)

First, the superolateral marking of the segmental defect angles was carried out (Fig. 1). Starting from the limbus acetabuli, a segmental defect angle of 40° was plotted superolaterally. The 80° and 120° segmental defect angles were each marked 20° medially and corresponding 60° and 100° superolaterally. The depth of the defect was about 5 mm. Three pigs each with Paprosky type I acetabular defects and without bone defects served as control groups. Then, all acetabula were milled out to a diameter of 38 mm. The bony defect sites were filled with the prepared cancellous bone that had been thawed to room temperature. Approx. 15 cm^3^ of cancellous bone was used (Fig. 1). The inserted bone was impacted using a 38 mm counterclockwise milling machine. After mixing the two cement components using a vacuum mixing system, the cementation was carried out using Refobacin Plus Bone Cement. Subsequently, the cemented implantation of a PE cup of the model Original M. E. Müller Low profile cup (Zimmer) with a diameter of 36 mm was carried out (Fig. 1). The three porcine pelvic bones that were not subject to defect milling of the acetabulum were merely reamed to 38 mm and received a cemented 36 mm cup.

### Biomechanical measurements

The biomechanical load parameters were measured using INSTRON type 8874 H 1003, which is a servo-hydraulic push and pull machine (Fig. 1). It has a measuring range of 10kN for pressure measurements and 100Nm for torque measurements. All 15 porcine hemipelves were clamped in a deflectable vice of the biotesting machine. The biomechanical force was transmitted with the aid of an MRP-TITAN stem implant, prosthetic neck without fin in size S from Brehm, at an angle of approx. 45° to the acetabular plane.

The biomechanical measurement data were processed with the aid of the program FT. StartUP V.7.22.

### Pressure analysis

**Fig. 2:**
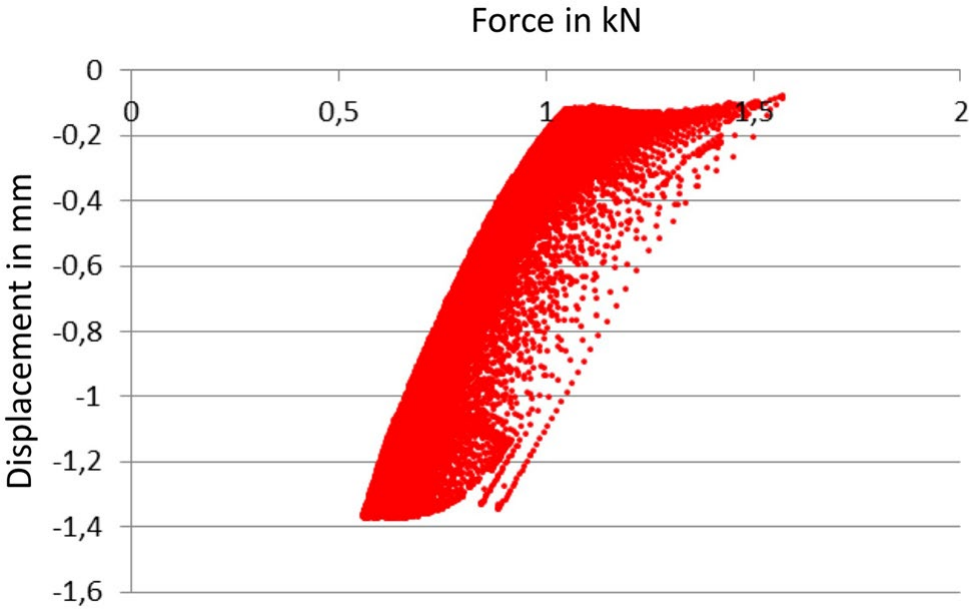
Force–displacement diagram of pressure analysis reflecting migration

The cup migration was initially determined as a change in path in the sense of a deformation after 1000 cycles and 2500 cycles. Then the difference was calculated from the two values, which reflect the migration of the cup in mm (Fig. 2).

To measure the compressive rigidity, a force of 1500 N was exerted on the pelves in 2500 cycles (1.6 s per cycle), corresponding to two and a half times the body weight of a person weighing 60 kg.

### Torsion analysis

This method was used to measure the stiffness, stability and resistance of the 15 porcine hemipelves. A metallic 22 mm femoral head, which was attached to an axial punch, was used to transmit the pressure. This femoral head was bonded to the acetabulum at an angle of 45° using Orthocryl sealing resin (Otto Bock, Duderstadt, Germany). This was followed by 2500 cycles with a constant axial preload of 0.5kN and an alternating torque of ± 0.6 Nm. The torque acting on the acetabulum and the associated angle φ were measured.

### Breakout analysis

In the breakout attempt, the PE cup was provoked to break out of the cement layer or the cancellous cement layer by increasing the torque. The maximum breakout moment and the breakout angle were recorded. There was an axial force transmission of a punch with a screwed-on femoral head.

### Statistical analysis

The statistical analysis was carried out using SPSS V.19. The measured values were compared in pairs with the control group without bone defects, using the Mann–Whitney *U* test for independent samples. The level of significance was set at 0.05. Mean values and standard deviations were calculated for all measured values.

## Results

**Fig. 3:**
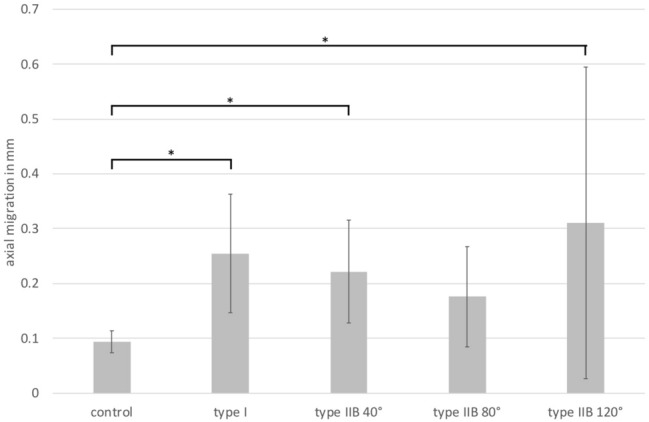
Axial cup migration after 2500 simulated gait cycles with 2.4kN load (*p < 0.0001)

After 2500 axial loading cycles, all cups in the deficient acetabula showed a typical asymptotic migration pattern in the sense of seating of the cup in the cancellous bone by approx. 0.26 mm ± 0.11 mm (Fig. 3). This results from the fact that the migration was significantly lower by about 0.1 mm in the primary cemented acetabula (*p* < 0.001 in all cases). No differences in the extent of migration were detectable between the different defect sizes.

**Fig. 4:**
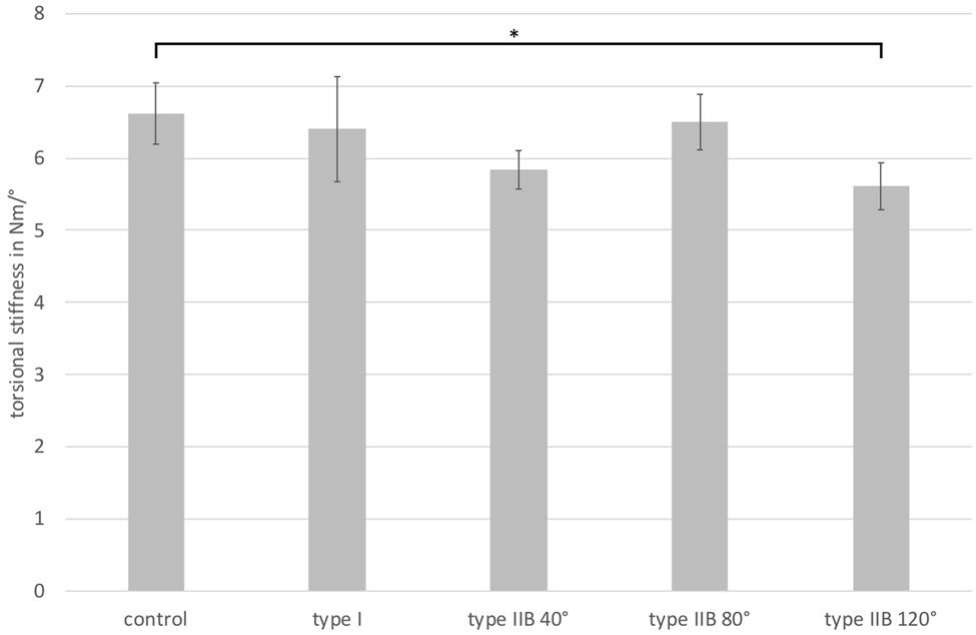
Torsional stiffness in Nm/° with an axial load of 0.5kN and a cyclic torsion moment of 0.6Nm (*p < 0.0001)

Torsional stiffness did not differ between the control group without defects, Paprosky type I defects and type IIB defects up to segmental defect angles of 80°. The torsional stiffness decreased significantly at segmental defect angles of 120°, compared to cups without defects (Fig. 4).

**Fig. 5:**
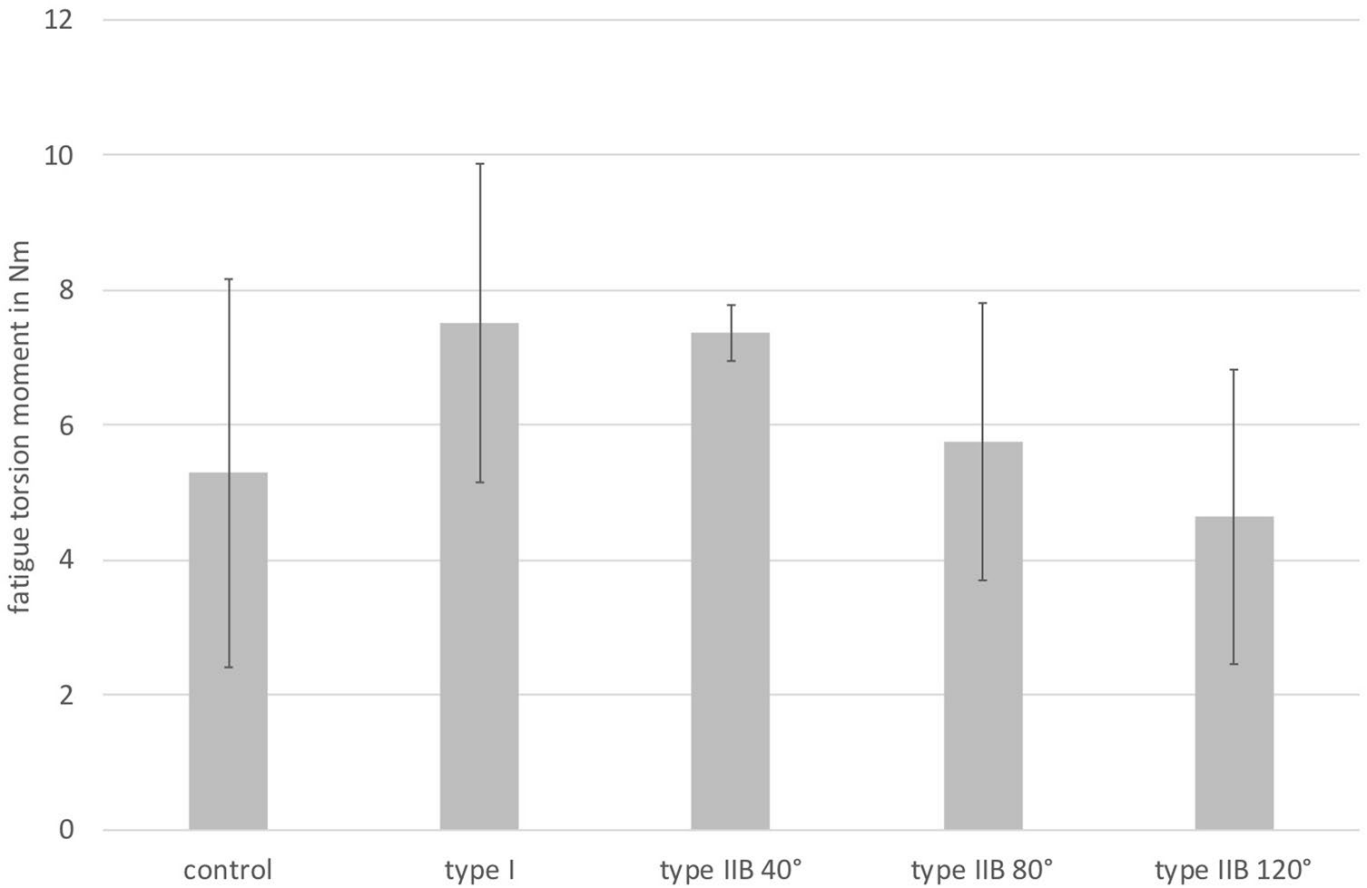
Torque until failure in Nm (*p < 0.0001)

This was followed by torque increase up to the rupture of the cups. Owing to the relatively large scatter between the individual specimens, there was a considerable standard deviation (Fig. 5). This did not reveal any group differences in the maximum torque until failure.

The ANOVA revealed a significant intergroup difference for the construct migration when comparing all groups (*p* = 0.05) which was not significant in the post hoc Tukey’s test (*p* = 0.103). Neither the ANOVA nor the Tukey’s test showed a significant intergroup difference for the torsional stiffness (*p* = 0.069 and *p* = 0.102). Also, the maximum rupture torque was not different between the groups when comparing all of them using the ANOVA and post hoc Tukey’s test (*p* = 0.219 and *p* = 0.450).

## Discussion

The main result of the present study is that IBG with Paprosky IIB defects up to a segmental defect angle of 80° achieves the same biomechanical primary stability as IBG with type I defects. Up to now, IBG has only been used to treat segmental defects in combination with additional wire mesh [1].

The intention is to transform the segmental defect into a cavitary defect. Long-term results with a revision rate of only 6% after 13 years in patients under 50 years of age have been achieved using IBG [9]. In contrast, Paprosky type III defects show a high loosening rate of the cup of 15% after 12.4 years based on radiological criteria [10]. Even if wire mesh is used, defect size seems to limit the use of IBG.

The insertion of wire meshes into the area of segmental defects requires the additional detachment of muscles, which, in addition to direct muscle trauma, can also lead to denervation damage to the pelvitrochanteric muscles, due to damage to the variable superior gluteal nerve [11]. In addition, the size of the cancellous bone used is also critical, as small pieces of cancellous bone show greater early migration and thus lead to poorer primary stability than large pieces of cancellous bone [12]. Large bone grafts, however, have a poor revascularization at the poorly perfused acetabular roof, resulting in biomechanical failure in most cases.

Metallic augments and support shells have become established in the treatment of segmental bone defects [1, 13], whereby metallic augments are primarily inserted without cement [14]. Supporting shells are screwed in place, converting segmental defects into cavitary defects, and enlarge the contact area with the autochthonous bone without integration into the bone [15–17]. Despite the good results of metallic solutions and the simpler surgical technique compared to IBG, IBG has a significant advantage. Ullmark et al. were able to show that, within the first postoperative months, revascularization of corticocancellous chips begins with the formation of woven bones, which leads to a remodeling into vital bone [15, 18]. Thus, after IBG, 45 re-revisions showed a significantly lower Paprosky acetabular defect [19]. Such de-escalation of bone defects appears to be particularly relevant in the increasing revision burden of biologically younger patients.

In 1996, Sloof et al. used washed pieces of cancellous bone with a size of 7–10 mm, wire mesh and cement to evaluate the clinical outcome of acetabular defects by means of radiographic measurements [20, 21]. Peripheral and medial segmental defects/cup defects were converted into cavitary acetabular defects by installing a wire mesh, and a wire mesh was placed between the impacted cancellous bone layer and the cup [20, 21]. This technique turned out to be beneficial, because long-term results were achieved with a revision rate of only 6% after 13 years in patients under 50 years of age [9]. However, in biomechanical measurements they found that small pieces of cancellous bone caused greater cup migration when an axial pressure of 1.5 kN was applied than large pieces of cancellous bone [12]. On the other hand, our biomechanical load measurements demonstrate that medium-sized cancellous chips of 5 mm, which were washed and dried, resulted in optimal cup stability for segmental defect angles up to 80° for Paprosky type IIB defects. The great advantage of medium-sized cancellous chips is the closer adaptation to the irregular acetabular defects, without gap formation.

In contrast to Sloof et al. we carried out three centrifugations, including two hours of air drying in order to remove all liquids from the cancellous chips. In combination with layer-by-layer compaction of the cancellous bone chips using a counterclockwise milling machine of 38 mm using the Exeter technique, this prevented subsequent cup migration due to volume expansion of the cancellous bone chips.

In 2021, Garcia-Rey et al. showed that the technique of IBG in combination with cemented cups, which we also used, achieved a reduced re-revision rate of just 9.6% over a follow-up period of 9 years [19]. In this study, bone stock augmentation by IBG showed longer implant survival [19]. The combination of IBG and acetabular cementation could have led to permanent secondary stability through bone integration and thus to longer implant survival. It can be assumed that the use of IBG results in better infiltration of the cement into the cancellous bone chips, which results in a thinner cement layer, but better interlocking, promoting bone ingrowth into the prosthesis.

This results in greater surface contact with vital local bone, which could lead to the formation of braided bones through postoperative revascularization, better mechanical stability and remodeling into vital bone over a period of years [15, 18, 22].

Confirming this, Butscheidt et al. showed in 2021 in a postmortem analysis of human acetabula that impacted allograft cancellous bone chips demonstrated osseous integration in 91.3% after an in situ retention time of 10.3 ± 4.5 years. The ingrowth and increased trabecular density of the allograft cancellous bone chips were detected by HR-pQCT, histologic analysis and scanning electron microscopy [23].

In addition, the results of our rupture test show that the use of IBG in Paprosky type I acetabular defects achieved significantly greater implant stability, as compared to defect-free acetabula without IBG.

The present study shows that IBG achieves sufficient primary stability in Paprosky IIB defects up to a segmental defect angle of 80° even without additional wire meshes or metallic augmentations.

Critical to the methodology we used is that there was no measurement of the long-term stability of bone defect reduction by IBG.

Thus, osseous integration of cancellous bone chips was not demonstrated by evidence of bone consolidation.

This may be critical, especially in the main loading zone of the segmental defect.

Despite the similarity in macro- and microscopic structure of porcine and human hips, different acetabular loading is present. This is due to the quadrupedal gait.

Therefore, further investigation is required before clinical recommendations can be made.

## Conclusion

Impacted cancellous bone-cement PE cups with Paprosky type IIB acetabular defects up to a defect angle of 80°, lacking metal augmentation, are biomechanically equivalent to defect-free cups.

It should be emphasized that the composite system of cancellous bone–cement–PE cup achieves significantly better biomechanical stability in Paprosky type I defects compared to the defect-free cup without IBG with cemented cup.
